# The Safety of Ethnic Foods in Campania Region: A Preliminary Study

**DOI:** 10.3390/foods12061244

**Published:** 2023-03-14

**Authors:** Loredana Biondi, Andrea Fulgione, Assunta De Lella, Anna Cutarelli, Morena Nappa, Francesca Garofalo, Stefania Cavallo, Donatella Nava

**Affiliations:** 1 Department of Food Security Coordination, Istituto Zooprofilattico Sperimentale del Mezzogiorno, 80055 Portici, Naples, Italy; 2 Department of Epidemiologic and Biostatistic Regional Observatory (OREB), Istituto Zooprofilattico Sperimentale del Mezzogiorno, 80055 Portici, Naples, Italy

**Keywords:** ethnic foods, food safety, food labeling, allergens

## Abstract

Ethnic food is produced by an ethnic group—using their familiarity with local ingredients of plants and/or animal origin—and it is consumed in a country other than the country of origin. In Italy, the ethnic food market has expanded over the last three decades. The aim of this study was to evaluate the correct labeling, the microbiological communities and the allergens present in 50 ethnic foods. The visual inspection of labels and microbiological and allergen analyses have been carried out for evaluating their food safety. The visual inspection of labels revealed the absence of labeling in Italian and/or a failure to specify the place of origin. Microbiological analyses showed the absence of pathogens (i.e., *Salmonella* spp., *Listeria monocytogenes* and *E. coli* 0157:H7) in all matrices, but the presence of process hygiene indicator bacteria (total bacterial count, Coagulase-positive Staphylococci, *Bacillus cereus*, coliforms, yeasts and molds) was found in 37 samples. With regard to allergens, 12 samples were non-compliant for the presence of at least one allergen, while only two products were of species different from those declared on the label. This research highlights the need to increase the control of ethnic foods and also to improve the labeling system by standardizing international regulations.

## 1. Introduction

The term ethnic food refers to a specific product consumed in a country other than the country where it is produced, and with intrinsic characteristics which make it unusual, different or new for consumers [1]. Universally, ethnic food can be considered as an ethnic group’s or a country’s cuisine that is culturally and socially recognized by consumers of other ethnic groups [2].

Ethnic foods include ready-to-eat meals, sauces, snacks, fish- and meat-based foods, and ingredients such as spices and herbs, etc. Several factors, including the influence of foreign cultures on local cuisines and the rising number of ethnic restaurants, are favoring the internationalization of several ethnic cuisines, such as Indian, Italian, Chinese, Mexican and Japanese. In this context, ethnic food cannot be regarded only as a mixture of specific ingredients, but also as a way of seasoning, cooking or consuming food, making the final product representative of a specific ethnic population [3].

In Europe, the demand for ethnic food is growing and heterogeneous. In some countries such as Belgium or France which have been exposed to immigration, research has focused on the analysis and characterization of ethnic foods [4,5,6]. In other countries, such as those of the northern shores of the Mediterranean, research has developed more slowly [3]. Immigration, tourism and globalization have highlighted the significant influence of ethnicity not only on business and consumer behavior but also on food culture and on food industry. At the same time, the increased consumption of ethnic foods has facilitated the spread of several food-borne illnesses [7].

Annually, about two million people worldwide die after eating unsafe food [8]. More than 200 food-borne diseases are caused by pathogens (such as bacteria, parasites, etc.) or chemical agents (such as toxins, allergens, pesticides, etc.).

Regarding pathogens, during the last ten years, cases of *Salmonella* spp. and *Listeria monocytogenes* (*L. monocytogenes*) have been associated with the consumption of sushi rolls in Brisbane and Hispanic-style soft cheese in the USA. Furthermore, a cluster of thirteen confirmed and eight suspected cases of *Escherichia coli* O157 (*E. coli* 0157) were associated with the consumption of food at an Indian restaurant in the United Kingdom.

Regarding allergens, cases of undeclared gluten in dried egg noodles and barley grass have been reported in several European countries, including Italy and the United Kingdom. Another episode involving the presence of a food allergen was reported after the consumption of cumin containing traces of peanut.

Increasing migration will favor the introduction of ethnic foods into the diet of both local and migrant populations. Thus, there is an urgent need to evaluate and prevent the presence of pathogens and/or allergens in ethnic foods [8]. In Italy, immigration has increased over the last three decades, with the number of immigrants reaching approximately 5 million as of 1 January 2020; this influx has modified the social and economic status, lifestyle and eating habits of the whole population [9,10]. The Italian market of ethnic foods is expanding; in fact, there was a growth of 15.4% in the consumption of ethnic foods in 2020 compared to 2019, as revealed by the national report COOP 2020 [11]. This trend reflects the increase in dining out, tourist flows between different countries and the increased demand for new foods.

The increased consumption of ethnic foods needs to be accompanied by an efficient traceability system [12]. Although the flow of immigrants into Italy and the diffusion of ethnic foods among the population are constantly growing, there is a lack of information on the composition and safety of ethnic foods [13]. Indeed, the ethnic food market presents several shortcomings regarding hygiene conditions, labeling and self-regulation. For example, the control and identification of these products are sometimes difficult owing to mislabeling and/or other illegal practices (intentional substitution or misrepresentation of food); both of which are defined as “economically motivated adulterations (EMA)” or, commonly, “food frauds” [14,15,16]. In other cases, even though the nutritional and microbial characteristics of several ethnic foods are well described, their safety needs to be investigated.

Based on the above considerations, the aim of this study was to evaluate the microbiological communities and allergens present in various ethnic foods and the correctness of product labeling, both of which are important for the safety of consumers.

## 2. Materials and Methods

### 2.1. Sampling and Label Inspection

**Figure 1:**
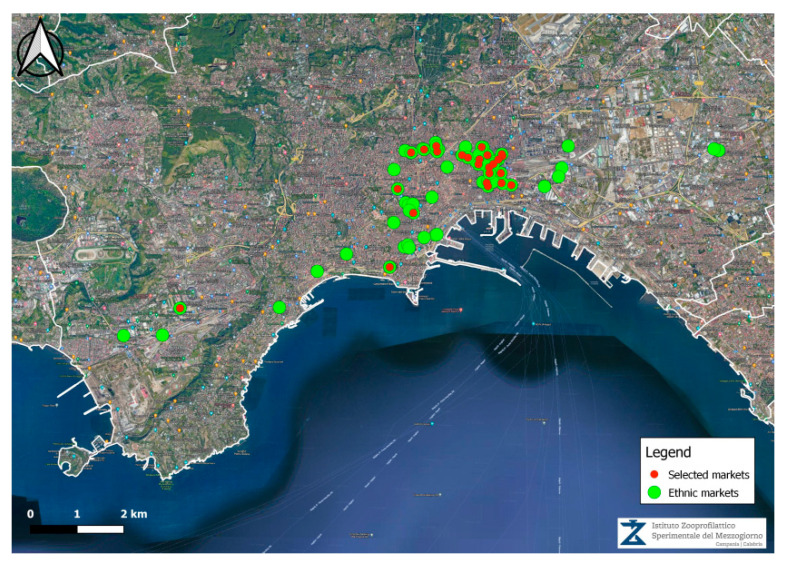
Map of the 72 ethnic markets (green dots) and of the 25 markets selected (red dots).

Samples were bought at local ethnic food retail outlets in the Neapolitan area, in the Campania region, by the local veterinary services of Napoli 1 Centro. All of these outlets were registered on GISA (Integrated Management of Services and Activities), a system developed by the Campania Region, which records various information on food retailers, such as the type of food products and the geographic position of the retail outlet. The above ethnic market consists of shops selling ethnic products, in accordance with the European Regulation (EC) No 852/2004. From the list of 72 ethnic food outlets, 25 were selected by means of a simple random sampling technique without repetition. Their geographic positions (Figure 1) were plotted by the Osservatorio Regionale Sicurezza Alimentare (ORSA) of the Istituto Zooprofilattico Sperimentale del Mezzogiorno by means of the software QGIS 3.22 (http://qgis.osgeo.org accessed on 22 October 2021).

From each market, two food samples were randomly selected according to their availability and were grouped into solid, liquid, spices and meat- or fish-based foods (Table 1).

The first step involved checking the labels. Specifically, the accuracy of the information reported on the label of each product was investigated to identify any non-conformity. This analysis was carried out according to [17], which refers to the labeling of fresh, frozen, dried, chilled, salted, smoked and pickled products, and not to cooked or prepared foods.

### 2.2. Bacteria

The detection of pathogens (*L. monocytogenes*, *Salmonella* spp., *E. coli* O157:H7) and the counting of process hygiene indicator bacteria (total bacterial count, Coagulase-positive Staphylococci, *Bacillus cereus*, coliforms, yeasts and molds) were carried out according to the official ISO methodologies or specific reference methods and were compared with the limits of detection (microbiological criteria) reported in Table 2. If the microbiological criteria were not reported in European or national regulations [18,19], the guidelines for the risk analysis of food microbiology [20] were adopted. Given the nature, type and difficult collocation of some samples in specific food categories, the threshold values were set in accordance with the precautionary principle, whereby the lowest value is regarded as the limit of detection (LOD).

### 2.3. Allergens

The presence of allergens, according to European Regulation (EC) No 1169/2011, was evaluated in accordance with specific internally approved and validated methods by means of a specific kit: “Ridascreen” (R-Biopharm Italia srl, Melegnano (Milan), Italy), as reported in Table 3. The analyses were carried out according to the kit manufacturer’s protocol.

### 2.4. Detection of Bacillus cereus Enterotoxin Genes

The detection of the three genes encoding for cytotoxins haemolysin BL (Hbl; codified by *hblC*, *hblD* and *hblA*), nonhemolytic enterotoxin (Nhe; codified by *nheA*, *nheB* and *nheC*) and cytotoxin K (cytK; codified by *cytK*) was carried out by means of Multiplex PCR.

In particular, while cytK consists of only one protein, both Hbl and Nhe are composed of three protein components. Hbl is constituted of two lytic elements L2 and L1, and a binding component B, which are encoded by *hblC*, *hblD* and *hblA*, respectively. Nhe is also composed of two lytic elements NheA and NheB, and the NheC protein with unknown function, which are encoded by *nheA* and *nheB*, and *nheC*, respectively [21].

Briefly, 4–6 colonies of *B. cereus* were suspended in 2 mL of nutrient broth (Merck Life Science S.r.l., Milan, Italy) and the mixture was centrifuged at 12,000× *g* for 5 min. The pellet was washed with 1 mL of sterile MilliQ water and centrifuged at 12,000× *g* for 5 min. It was then re-suspended in 300 µL of Chelex 100 (Merck Life Science S.r.l., Milan, Italy), incubated at 56 °C for 20 min and boiled (at 100 °C) for 8 min. Subsequently, it was cooled in ice for 10 s and centrifuged at 12,000× *g* for 5 min (home-made protocol). Finally, 200 µL of supernatants was collected and stored at −20 °C until use. The multiplex PCR assay was carried out as reported [22]. The amplicons were separated using the QIAxcel Advanced System (Qiagen GmbH, Hiden, Germany). *B. cereus* ATCC 14579 was selected as the reference strain.

### 2.5. Identification of Fish and Meat Species

DNA extraction and sequencing were carried out for identifying and confirming the fish and meat species of the products with those declared on the label. Briefly, DNA was extracted from 25 mg of fish or meat samples by means of a QIAamp DNA Mini kit (Qiagen GmbH, Hilden, Germany). The target of the analysis was the gene *cytB* (around 360 bp) of mitochondrial DNA, which was amplified using primer CYTB1 [23]. A PCR reaction mix (50 µL) included 5 µL of DNA (100 ng), 25 µL HotStar Taq Master mix (Qiagen GmbH, Hilden, Germany), 1 µL of each primer (10 µM) and 18 µL of RNAse/DNAse-free water (Merck Life Science S.r.l., Milan, Italy). Thermal profiling consists of the following steps: 15 min at 95 °C, 40 cycles of 15 min at 95 °C, 30 s at 48 °C, 1 min at 72 °C, and a final elongation step of 7 min at 72 °C. The PCR was carried out using T100 Thermal Cycler (Bio-Rad Laboratories, Hercules, CA, USA). Amplicons were separated by means of the QIAxcel Advanced System (Qiagen GmbH, Hilden, Germany).

For sequencing, PCR products were purified by means of Exosap-IT (Applied Biosystems, Foster City, CA, USA) and bi-directionally sequenced using the BigDye Terminator v1.1 Cycle Sequencing Kit (Applied Biosystems, Foster City, CA, USA), according to the manufacturer’s recommendations. Sequences were dye-terminator-removed by means of Edge-Bio (Edge BioSystems, San Jose, CA, USA) and then run on Seqstudio (Applied Biosystems, Foster City, CA, USA). Electropherograms were analyzed using sequencing analysis v5.2 (Applied Biosystems, Foster City, CA, USA). The sequences obtained were compared with those reported in BLAST [24].

## 3. Results

This research aimed to provide preliminary results concerning the safety of ethnic foods. The visual inspection of labels revealed that some products showed non-conformity in labeling. Specifically, 14 products did not conform: 6 were not labeled in Italian, 5 did not indicate the place of origin and 3 did not conform with either parameter (Table 4).

Microbiological analyses revealed the absence of the following pathogens in all matrices: *Salmonella* spp., *L. monocytogenes* and *E. coli* 0157:H7. Regarding the other microbiological parameters, 13 food samples conformed with the microbiological criteria, while the remaining 37 did not conform with at least one parameter. Specifically, 28 cases involved the TBC, 6 involved coagulase-positive Staphylococci, 8 involved molds, 6 involved *B. cereus* and 5 were positive for the presence of coliforms (which indicate deficient hygiene). Notably, 13 food matrices did not conform with at least two microbiological parameters (Table 5).

On the basis of the microbiological results, the matrices displayed contamination in descending order, as follows: spices, fish-based products, liquid matrices, solid matrices and meat-based products.

**Figure 2:**
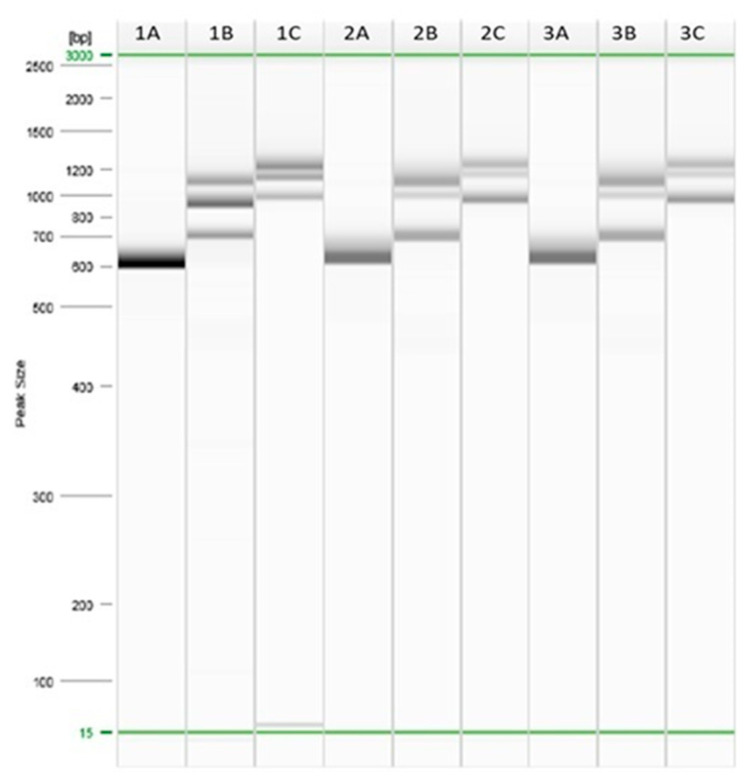
Multiplex PCR of *Bacillus cereus* in the following samples: (A) grain anise; (B) fenugreek and (C) instant noodles. The letter in each column indicates (A) *cytK* gene, (B) *nhe* genes and (C) *hbl* genes.

In addition, the *B. cereus strains* isolated from samples were characterized for the presence of enterotoxin genes via multiplex PCR. The results have shown the presence of the enterotoxin genes in three samples: grain anise, fenugreek and instant noodles, as reported in Figure 2. In particular, the results have shown the presence of enterotoxin gene fragments of about 582, 657, 858, 996, 922, 1113 and 1245 bp, for *cytK*, *nheC*, *nheA*, *nheB*, *hblC*, *hblA* and *hblD* genes, respectively. All of these genes belong to a virulence region and are considered to be the principal genes responsible for the strain’s pathogenicity [25].

Regarding allergens, only eight samples were non-compliant with regard to the presence of one allergen, and only four samples contained two undeclared allergens (Table 6).

In total, of the 50 ethnic foods analyzed, 37 did not respect microbiological standards, 12 contained undeclared allergens and 8 displayed both of these defects (Table 7).

Regarding the identification of fish and meat species, the use of DNA barcoding allowed us to characterize some of the fish- and meat-based foods. The results confirmed that the species was declared on the label in six cases, while two products were of species that did not comply with those declared on the label (lizardfish and Eastern monkfish fillets) and the remaining one (smoked prawns) was not identified (Table 8).

## 4. Discussion

The label of a food product can be regarded as its “ID card”; it therefore constitutes an important element in food commercialization and traceability. In Europe, EC Regulation No 1169/2011 [17] states that all components (including allergens) must be declared on the label of all foods distributed in the EU. Language barriers make it very difficult to label ethnic foodstuffs correctly, and some producers use this trick in order to elude specific types of food inspection. A failure to declare ingredients or allergens and the incorrect translation of commercial denominations constitute a danger for intolerant or allergic consumers.

On the other hand, it is not mandatory to declare on the label the possible presence of allergens due to accidental cross-contamination or cross-contact with allergens during the production and distribution processes. Although these events are rare in foods produced in Europe, in non-European countries in which hygienic–sanitary conditions are inadequate and/or uncontrolled, such accidents may be more frequent; thus, “precautionary labeling” is important.

The variability in our microbiological results probably stems from the heterogeneity of the food matrices selected for the study. Despite the absence of pathogens, i.e., *Salmonella* spp., *L. monocytogenes* and *E. coli* 0157:H7, the data on the other microbiological parameters suggest an inadequate hygienic–sanitary condition in which the different matrices are produced and/or stored. This situation was widely ascertained in spices, which presented a high total bacterial load and a significant presence of molds.

It is important to highlight that spices generally grow in temperate and tropical territories such as China, India, Brazil, Mexico, etc. The particular conditions of production (i.e., poor hygienic conditions, humid and temperate climate) could increase the risk of total aerobic mesophilic bacteria and mold contaminations [26,27]. In particular, after harvesting, spices are subjected to drying in the sun, thus increasing the risk of bacterial contamination caused by dust and insects [28]. Several techniques such as irradiation, heat treatments inhibiting the bacteria and spoilage microorganisms, the utilization of good hygienic practices for the entire process of production and the testing of the end product represent the most important instruments [28].

In addition, the presence of *B. cereus* in certain foods (instant noodles, instant vermicelli, caraway, aniseed, fenugreek and spice mixture) and, in particular, the presence of genes encoding enterotoxins Hbl, Nhe and cytotoxin K in three of these matrices (instant noodles, aniseed and fenugreek) make these products potentially dangerous for consumers. Indeed, the cytotoxins Hbl, Nhe and cytK are considered to be the main etiological agents of food-borne *B. cereus* diarrheal disease [25].

With regard to the allergen analysis, the results have shown that eight samples contained almost one undeclared allergen while four samples contained two undeclared allergens.

The fish and meat species identification confirmed the correspondence of six products to the species declared on the label, while two products were of species different from those declared on the label and only one was not identified.

On the basis of these preliminary data, and considering the increasing consumption of ethnic foods, we can conclude that greater attention should be focused on controlling the ethnic food trade (from product importation to sale) and also on training and information programs for food business operators.

Moreover, further studies should include a broader range of ethnic foods, in order to provide food safety authorities, producers and consumers with more information about ethnic foods and their safety.

Finally, this research highlighted two important aspects of the ethnic market. First, it is important to improve the labeling system by standardizing international rules and regulations and making it obligatory to specify the ingredients on the label, in order to safeguard the health of the consumer. Second, there is a need to develop a panel of analyses (microbiological, allergens, molecular and chemical) able to guarantee food safety, thus protecting public health.

## Figures and Tables

**Table 1:** Classification of food matrices.

Matrix	Description	Quantity
Liquid	Thai sauce (anchovy), soy sauce, guacamole sauce, coconut extract, sweet tea	5
Solid	Noodles (x2), beef-flavored and soy sauce noodles, instant noodles, Ruchi Chanachur (barbecue snack), instant vermicelli, instant wheat-flour noodles, soy noodles, dehydrated seaweed, canned cabbage with mustard, bamboo shoots, millennium eggs, Piadin-brik, mung bean vermicelli, salted Chinese seaweed	15
Spices	Spices for roast pork (x2), caraway (cumin), ginger, Ras el Hanut, grain anise, fenugreek, mixed spices for fish, harissa (chili sauce), spice blend for chili, vegetable curry	11
Fish-based foods	Sardines, lizardfish (*Saurida tumbil*), smoked prawns (*Penaeus duorarum*), Eastern monkfish fillets (*Ommastrephes bartrami*), shrimp cloud (x2), Gourami (*Trichogaster lalius*), jack mackerel in tomato sauce (*Trachurus symmetricus*), sprat fish, anchovy snack, fried fish	11
Meat-based foods	Al-Raii (chicken meat meal), Rou song (pork meal), chicken with Thai sauce, curry and rice (x 2), pork preserves, spicy beef sausage, turkey meat mortadella, chicken meat mortadella	8

**Table 2:** Microorganisms, reference methods and limit of detection used in the microbiological analyses of spoilage, pro-technological and pathogenic microorganisms.

Microorganisms	Reference Methods	Microbiological Criteria (Limit of Detection)
*Salmonella* spp.	UNI ^1^ EN ^2^ ISO ^3^ 6579-1:2020	Commission Regulation (EC) No. 2073/2005
*L. monocytogenes*	UNI ^1^ EN ^2^ ISO ^3^ 11290-1:2017	Commission Regulation (EC) No. 2073/2005
*E. coli* O157:H7	UNI ^1^ EN ^2^ ISO ^3^/ST ^4^ 13136:2013	Commission Regulation (EC) No. 2073/2005
Total bacterial count (TBC)	UNI ^1^ EN ^2^ ISO ^3^ 4833-1:2021	Conferenza Stato-Regioni 212/16; Linee guida per l’analisi del rischio nel campo della microbiologia degli alimenti.
*E. coli* β-glucuronidase-positive	UNI ^1^ ISO ^3^ 16649-2:2001	Conferenza Stato-Regioni 212/16; Linee guida per l’analisi del rischio nel campo della microbiologia degli alimenti.
Coagulase-positive Staphylococci	UNI ^1^ EN ^2^ ISO ^3^ 6888-2:2004	Conferenza Stato-Regioni 212/16; Linee guida per l’analisi del rischio nel campo della microbiologia degli alimenti.
*B. cereus*	ISO ^3^ 7932:2020	Conferenza Stato-Regioni 212/16; Linee guida per l’analisi del rischio nel campo della microbiologia degli alimenti.
Yeasts and molds	UNI ^1^ ISO ^3^ 21527-1:2008 (aw ≥ 0.95) UNI ^1^ ISO ^3^ 21527-1:2008 (aw ≤ 0.95)	Conferenza Stato-Regioni 212/16; Linee guida per l’analisi del rischio nel campo della microbiologia degli alimenti.
Coliforms	UNI ^1^ ISO ^3^ 4832:2006	Conferenza Stato-Regioni 212/16; Linee guida per l’analisi del rischio nel campo della microbiologia degli alimenti.

^1^ (UNI) Italian National Unification; ^2^ (ENs) European norms; ^3^ (ISO) International Standards Organization; ^4^ (ST) technical specifications.

**Table 3:** Allergens and detection methods plus reference kits.

Allergen	Reference Method	Kit
Gluten	AOAC Ridascreen n. 120601	RIDASCREEN Gliadin—R7001
Beta lactoglobulin	MP/AL/016	RIDASCREEN FAST Beta Lactoglobulin—R 4912
Egg protein	MP/AL/014	RIDASCREEN FAST Ei/Egg Protein—R 6402
Crustacean	MP/AL/006	RIDASCREEN FAST Crustacean—R 7312
Peanut	AOAC RIDASCREEN FAST Peanut n. 030404	RIDASCREEN FAST Peanut—R 6811
Hazelnut	MP/AL/039	RIDASCREEN FAST Soya—R 7102

**Table 4:** Visual inspection of ethnic food labels.

Matrix	Product	Absence of Italian Labeling	Unreported Origin	Absence of Italian Labeling and Unreported Origin
Liquid	Sweet tea		X	
Solid	Mung bean vermicelli		X	
Spices	Spices for roast pork	X		
	Ginger		X	
	Ras el Hanut		X	
	Grain anise		X	
	Fenugreek			X
	Caraway (cumin)	X		
	Mixed spices for fish	X		
Fish-based foods	Lizardfish (*Saurida tumbil*)	X		
	Jack mackerel in tomato sauce (*Trachurus symmetricus*)			X
Meat-based foods	Al-Raii (chicken meat meal)			X
	Turkey meat mortadella	X		
	Chicken meat mortadella	X		

(X): non-conformity in labeling.

**Table 5:** Microbiological analyses of food matrices.

Matrix	Product	Total Bacteria Count (CFU/g or mL)	Coagulase-Positive Staphylococci (CFU/g or mL)	Yeasts and Molds (CFU/g or mL)	*B. Cereus* (CFU/g or mL)	Coliforms (CFU/g or mL)
Liquid	Thai sauce (anchovy)	<100	4600 *	<100/<100	<100	<100
	Soy sauce	5900 *	4100 *	<100/<100	<100	<100
	Guacamole sauce	590 *	<100	<100/<100	<100	<100
	Coconut extract	1200 *	<100	<100/<100	<100	<100
Solid	Beef-flavored and soy sauce noodles	5600 *	<100	<100/<100	<100	<100
	Noodles	1500 *	<100	<100/<100	<100	<100
	Instant noodles	<100	<100	<100/<100	120 *	<100
	Ruchi Chanachur (barbecue snack)	68,000 *	<100	<100/<100	<100	<100
	Instant vermicelli	<100	<100	<100/<100	130 *	<100
	Soy noodles	3400 *	<100	<100/<100	<100	<100
	Dehydrated seaweed	560 *	<100	<100/1500 *	<100	<100
	Canned cabbage with mustard	530 *	<100	<100/<100	<100	<100
	Piadin-brik	<100	<100	<100/13,000 *	<100	<100
	Mung bean vermicelli	520 *	<100	<100/<100	<100	<100
Spices	Spices for roast pork	5100 *	4100 *	<100/<100	<1000	<100
	Caraway (cumin)	<100	<100	<100/3000 *	1100 *	8500 *
	Ras el Hanut	4.9 × 10^5^ *	<100	<100/45,000 *	<1000	6200 *
	Grain anise	5100 *	<100	<100/<100	1070 *	<100
	Fenugreek	7000 *	<100	<100/<100	1120 *	<100
	Mixed spices for fish	4.9 × 10^5^ *	100 *	<100/1300 *	<1000	<100
	Harissa (chili sauce)	<100	330 *	<100/<100	<1000	<100
	Spice blend for chili	3.7 × 10^5^ *	<100	<100/<100	1250 *	<100
	Vegetable curry	2000 *	<100	<100/<100	<1000	2100 *
Fish-based foods	Sardines	1.7 × 10^8^ *	<100	<100/<100	<100	<100
	Lizardfish (*Saurida tumbil*)	4.7 × 10^7^ *	<100	<100/<100	<100	<100
	Smoked prawns (*Penaeus duorarum*)	6.1 × 10^7^ *	410 *	<100/<100	<100	4100 *
	Eastern monkfish fillets (*Ommastrephes bartrami*)	1.8 × 10^7^ *	<100	<100/<100	<100	<100
	Shrimp cloud	1.3 × 10^7^ *	<100	<100/<100	<100	<100
	Gourami (*Trichogaster lalius*)	<100	<100	<100/1100 *	<100	<100
	Sprat fish	1200 *	<100	<100/<100	<100	<100
	Anchovy snack	890 *	<100	<100/<100	<100	<100
	Fried fish	4.9 × 10^5^ *	<100	<100/<100	<100	<100
	Shrimp cloud	10^7^ *	<100	<100/<100	<100	<100
Meat-based foods	Al-Raii (chicken meat meal)	<100	<100	<100/1000 *	<100	<100
	Rou song (pork meal)	<100	<100	<100/1500 *	<100	<100
	Pork preserves	860 *	<100	<100/<100	<100	<100
	Turkey meat mortadella	2.1 × 10^3^ *	<100	<100/<100	<100	2900 *

(*): values exceeding the permitted microbiological limit values.

**Table 6:** Allergens present in food matrices.

Matrix	Product	Gluten	Beta Lactoglobulin	Egg Protein	Crustacean
Liquid	Sweet tea		X		
Solid	Instant noodles	X			
	Instant vermicelli	X			
	Instant wheat-flour noodles		X	X	
	Dehydrated seaweed		X		X
	Mung bean vermicelli		X		
Spices	Caraway (cumin)	X	X		
Fish-based foods	Lizardfish (*Saurida tumbil*)				X
	Fried fish	X	X		
Meat-based foods	Spicy beef sausage		X		
	Turkey meat mortadella			X	
	Chicken meat mortadella			X	

(X): presence of allergen.

**Table 7:** Products displaying both microbiological and allergenic non-conformity.

Matrix	Product	TBC (CFU/g or mL)	Yeast/ Mold (CFU/g or mL)	*B. cereus* (CFU/g or mL)	Coliforms (CFU/g or mL)	Gluten (ppm)	Egg Protein (ppm)	Βeta Lactoglobulin (ppm)	Crustacean (ppm)
Solid	Instant noodles	<10	<100/<100	**120**	<10	**63.90**	Not detected	Not detected	Not detected
	Instant vermicelli	<10	<100/<100	**130**	<10	**62.38**	Not detected	Not detected	Not detected
	Dehydrated seaweed	**560**	<100/**1500**	<100	<10	Not detected	Not detected	**0.965**	**62.69**
	Mung bean vermicelli	**520**	<100/<100	<100	<10	Not detected	Not detected	**0.850**	Not detected
Spices	Caraway (cumin)	<10	<100/**3000**	**1100**	**8500**	**62.58**	Not detected	**0.260**	Not detected
Fish-based foods	Lizardfish (*Saurida tumbil*)	**4.7 × 10^7^**	<100/<100	<100	<10	Not detected	Not detected	Not detected	**116.92**
	Fried fish	**4.9 × 10^5^**	<100/<100	<100	<10	**68.41**	Not detected	**0.307**	Not detected
Meat-based foods	Turkey meat mortadella	**2100**	<100/<100	<100	**2900**	Not detected	**1.07**	Not detected	Not detected

**Table 8:** Multiplex PCR of meat- and fish-based foods.

Products	Declared Species	Identified Species
Sprat fish	*Sprattus sprattus*	*Sprattus sprattus*
Al-Raii (chicken meat meal)	*Gallus gallus*	*Gallus gallus*
Sardines	*Sardinia philcardus*	*Sardinia philcardus*
Lizardfish	*Saurida tumbil*	*Saurida umeyoshii*
Smoked prawns	*Penaeus duorarum*	*Not identified*
Eastern monkfish fillets	*Ommastrephes bartrami*	*Larimicht hyspolyactis*
Anchovy snack	*Engraulis japonicu*	*Engraulis japonicu*
Rou song (pork meal)	*Sus scrofa*	*Sus scrofa*
Millennium eggs	*Anas platyrhynchos*	*Anas platyrhynchos*

## Data Availability

The data are available from the corresponding author.
